# Energy dependence of the GAFCHROMIC LD‐V1 in the diagnostic radiographic modalities

**DOI:** 10.1002/acm2.70117

**Published:** 2025-05-09

**Authors:** Tatsuhiro Gotanda, Tomoyuki Hasuo, Shinnosuke Nishihara, Kohsei Matsuura, Yasuyuki Kawaji, Hidetoshi Yatake, Shinya Imai, Takuya Akagawa, Nobuyoshi Tanki, Toshizo Katsuda

**Affiliations:** ^1^ Department of Radiological Technology, Faculty of Health Science and Technology Kawasaki University of Medical Welfare Okayama Japan; ^2^ Department of Radiological Science, Faculty of Health Sciences Junshin Gakuen University Fukuoka Japan; ^3^ Department of Breast Cancer Center Kaizuka City Hospital Osaka Japan; ^4^ Department of Radiological Science, Faculty of Health Science Morinomiya University of Medical Sciences Osaka Japan; ^5^ Department of Radiological Technology Tokushima Red Cross Hospital Tokushima Japan; ^6^ Brain Activity Imaging Center ATR‐Promotions Inc. Kyoto Japan; ^7^ Department of Medical Radiation Sciences Shizuoka College of Medicalcare Science Shizuoka Japan

**Keywords:** diagnostic radiographic modalities, energy dependence, GAFCHROMIC LD‐V1

## Abstract

The GAFCHROMIC LD‐V1 radiochromic film is widely used in dosimetry because it can provide high‐resolution two‐dimensional dose distributions without processing. This study aimed to evaluate the response characteristics at different effective energies, from the low‐energy range of mammography to the high‐energy range of computed tomography. Net pixel value (NPV)‐absorbed dose calibration curves for the GAFCHROMIC LD‐V1 were generated using x‐rays with effective energies of 18, 30, 50, and 80 keV to reflect those used in different diagnostic radiographic modalities. The film response was analyzed using calibration curves at each energy level. The coefficients of determination for the calibration curves at 18, 30, 50, and 80 keV were 0.9992, 0.9997, 0.9999, and 0.9976, respectively. The pixel value change at 30 keV was the largest and most sensitive, while the smallest change in pixel value and lowest sensitivity were noted at 18 keV. Because the energy dependence of the GAFCHROMIC LD‐V1 is significant below 18 keV and above 80 keV, it is necessary to establish an appropriate NPV‐absorbed dose calibration curve for energies below 18 keV and consider the possibility of underestimating the dose at energies above 80 keV.

## 1 INTRODUCTION

Radiochromic films offer a simple approach to measuring absorbed doses without needing developmental processing, as their pixel value changes in response to dose changes. In addition, they provide high‐resolution, two‐dimensional dose distributions applicable to various imaging modalities.^1^, ^2^, ^3^, ^4^, ^5^, ^6^, ^7^, ^8^

There are two main types of radiochromic films: those used in radiotherapy and those used in diagnostic radiology; the active layer of the two film types differs in composition. Radiochromic films used for radiotherapy are composed of tissue‐equivalent materials, resulting in a low effective atomic number and minimal energy dependence. In contrast, radiochromic films used for diagnosis are composed of materials with highly effective atomic numbers to enhance sensitivity, leading to significant energy dependence that must be considered.^1^, ^9^, ^10^, ^11^ Radiochromic films have been widely used in clinical settings for dosimetry applications due to their high spatial resolution, self‐developing properties, and ease of use. In radiotherapy, they are commonly employed for quality assurance and patient‐specific dose verification in external beam therapy and brachytherapy. In diagnostic radiology, they are utilized for entrance skin dose measurement, x‐ray beam profile analysis, and patient dose assessment in interventional radiology procedures.

Among radiochromic films, GAFCHROMIC LD‐V1 (LD‐V1: Ashland Inc., USA) is a newly developed radiochromic film with high sensitivity to low‐dose x‐ray exposure, suggesting its potential applicability in dose evaluation for mammography and computed tomography (CT).^12^ In these modalities, ionization chambers are widely used for dose measurements. In mammography, they are employed to evaluate the mean glandular dose, which typically ranges from 1 to 3 mGy per exposure.^12^, ^13^ In CT, pencil‐type ionization chambers measure the computed tomography dose index, with typical values ranging from 10 to 50 mGy for routine CT and approximately 100 mGy for high‐energy CT applications.^13^, ^14^ Although LD‐V1 has not yet been fully established for dose evaluation in mammography and CT, its sensitivity to low doses suggests its potential as a valuable tool for dosimetry in diagnostic radiology. It is sensitive to doses ranging from 20 to 200 mGy and covers an energy range of 40–160 kVp, according to the manufacturer.^15^ However, a previous experiment demonstrated that measurements are possible at 1 mGy, which is lower than the recommended dose range.^16^ This suggests the potential for assessing patient radiation exposure across various diagnostic radiology modalities. Therefore, this study aimed to evaluate the response characteristics of the LD‐V1 by assessing its energy dependence across different effective energies, from the low‐energy range of mammography to the high‐energy range of CT. Understanding the energy dependence of LD‐V1 is essential for its clinical application in dosimetry, as it allows for more accurate dose measurements across different diagnostic radiology modalities.

## 2 MATERIALS AND METHODS

### 2.1 LD‐V1

The LD‐V1‐1012 film is rectangular, measuring 10 × 12 inches (25.4 cm × 30.5 cm), with a thickness of 239 µm. It consists of an orange polyester layer, a pressure‐sensitive adhesive layer, an active layer, and a white polyester layer.^15^ The image was acquired using a scanner in reflection mode. When the active component was exposed to radiation, its color changed to dark blue. The effective energy range of the LD‐V1 is 40–160 kVp, with a dosimetric range of 20–200 mGy.^15^ The LD‐V1 used in this study was stored in a light‐shielded bag at room temperature (20°C–25°C).

### 2.2 Energy response of the LD‐V1

#### 2.2.1 Calculation of effective energies

The LD‐V1 was irradiated with x‐rays of effective energies used in diagnostic radiographic modalities, including mammography, general radiography, interventional radiology, and CT. For mammography, the dosimeter was irradiated with tungsten (W)/rhodium (Rh) x‐ray beams at 28 kV and 10 mAs using a mammography machine (Mammomat 3000, Siemens, Germany). An ionization chamber (TN23344, 0.2 cm^3^ soft x‐ray chamber, PTW, Germany) was used as the dosimeter to measure the half‐value layer and determine the effective energy of the x‐ray beam prior to film irradiation. The half‐value layer (HVL) was calculated by creating an attenuation curve using an aluminum (Al) filter with over 99.9% purity and a soft x‐ray chamber (type TN23344). The soft x‐ray chamber (TN23344) was calibrated regularly by the manufacturer, ensuring traceability to national standards. Temperature compensation was automatically managed by the electrometer (EMF521R, PTW, Germany), while pressure corrections were applied using the Boyle–Charles law based on ambient conditions. These calibrations ensured that the measured exposure and dose values accurately reflected the true x‐ray output under standard conditions. W/Rh x‐ray beams are produced using a W anode, with the x‐rays filtered through a Rh filter. This combination is commonly used in mammography to generate a low‐energy x‐ray spectrum, which is ideal for imaging soft tissues such as the breast, providing high contrast. In accordance with guidelines by the European Reference Organization for Quality Assured Breast Screening and Diagnostic Services, a normal geometric arrangement was adopted for measuring the HVL to minimize the influence of scattered radiation.^17^ The dosimeter measurement surface was positioned at the center of the breast support table, 50 mm in height and 40 mm from the chest wall edge. Additionally, an Al filter was placed on the compression paddle and positioned as close to the x‐ray tube as possible. Exposure and dose measurements were obtained thrice for each Al thickness, with the average value being utilized. These calibrations ensured that the measured exposure and dose values accurately reflected the true x‐ray output under standard conditions. The effective energy was obtained from the HVL, which was converted to effective energy using recent data from Seltzer and Hubbell at the National Institute of Standards and Technology.^18^

For the other diagnostic radiographic modalities, an x‐ray generator (KXO‐32SS, Canon, Japan) was used with effective energies of approximately 30, 50, and 80 keV for general radiography, interventional radiology, and CT, respectively. In CT, the effective energy varies across the field of view due to beam‐hardening effects. Based on previous studies, an effective energy range of 50–80 keV was assumed for this study.^19^ According to those findings, the effective energy at the CT isocenter was approximately 50 keV, increasing to approximately 70 keV at a horizontal distance of 20 cm from the isocenter. This energy shift is expected to influence LD‐V1 sensitivity and should be considered when interpreting dosimetric measurements. The irradiation conditions of 30, 50, and 80 keV were set to 65 kV, 7.1 mAs (100 mA × 0.071 s); 120 kV + 4 mm Al, 4.0 mAs (50 mA × 0.08 s); and 120 kV + 2 mm Cu, 25.0 mAs (200 mA × 0.125 s), respectively. A farmer chamber (TN30013, 0.6‐cm^3^ Farmer‐type chamber, PTW, Germany) was used. As with the soft x‐ray chamber used for mammography, this Farmer‐type chamber was also calibrated regularly by the manufacturer and corrected for temperature and pressure variations using the same methods. An Al‐filtered attenuation curve was generated, and the exact effective energy was calculated from the HVL. The geometrical arrangement complied with the International Organization for Standardization and was set up such that the ionization chamber was not affected by scattered rays.^20^

### 2.3 LD‐V1 calibration curve

#### 2.3.1 Exposure methods

**FIGURE 1:**
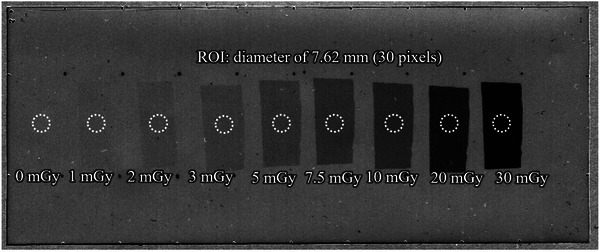
Scanned image of the LD‐V1 after exposure for calibration curve construction for mammography. Regions of interest (ROIs) on the LD‐V1 are shown as white circles with 30‐pixel diameters. LD‐V1, GAFCHROMIC LD‐V1.

The response characteristics of the LD‐V1 for each of the effective energies were investigated using the net pixel value (NPV)‐absorbed dose calibration curve of the LD‐V1 versus film pixel value. NPV quantifies the change in the red channel pixel values of the film before and after irradiation, serving as an indicator of radiation exposure. The detailed calculation of NPV is described in section C.2. To construct the NPV‐absorbed dose calibration curve, nine regions of the LD‐V1 sheet were exposed to a dose range of approximately 0–30 mGy (Figure 1). To eliminate errors from slight variations in the light source or scanning position during repeated scans, multiple regions were exposed to different doses on a single sheet of film.

**FIGURE 2:**
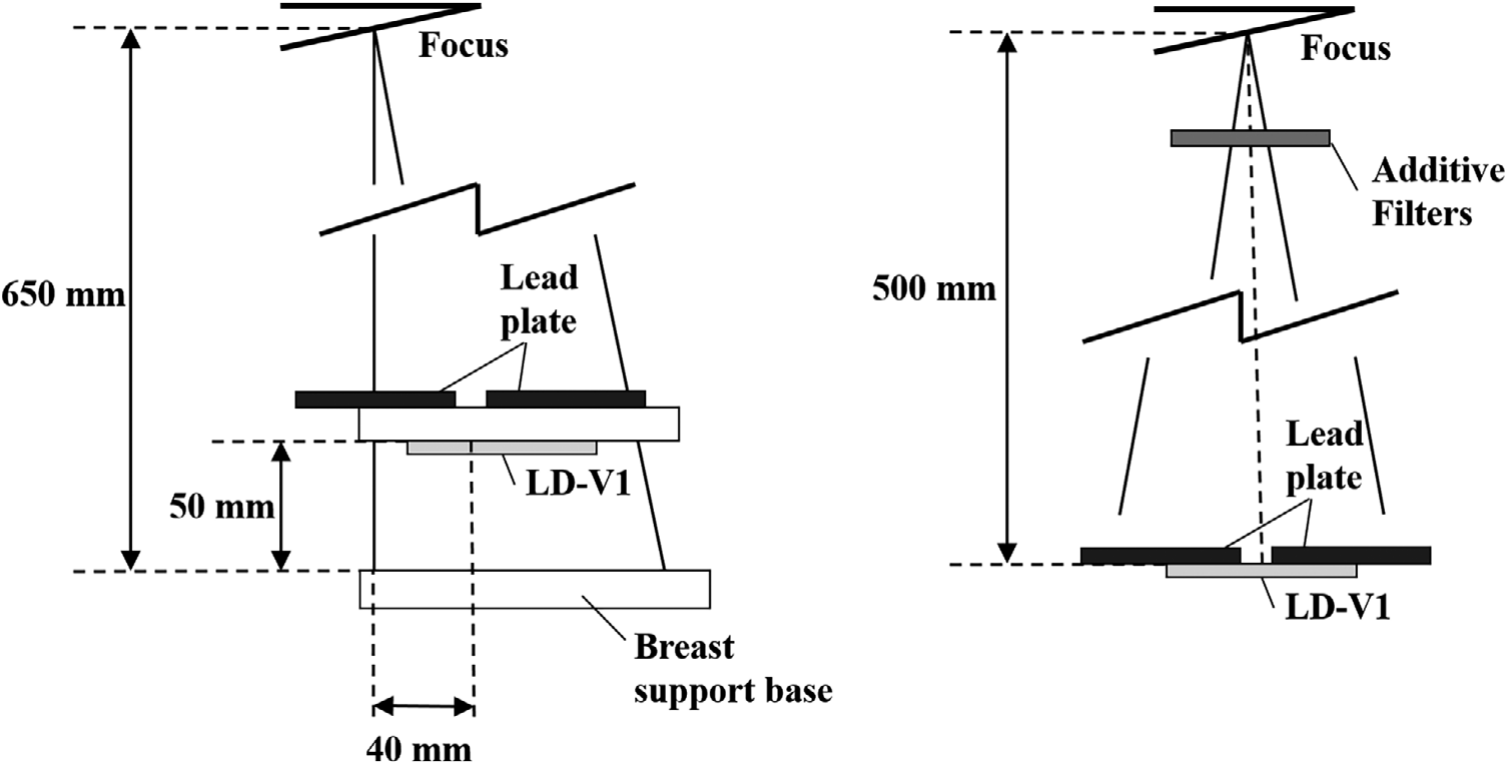
Graphical representation of the LD‐V1 irradiation apparatus. The distance from the x‐ray focus to the LD‐V1 was 600 and 500 mm for (a) mammography and (b) the other diagnostic radiographic modalities, respectively. LD‐V1, GAFCHROMIC LD‐V1.

The irradiation field size was 10 cm^2^ × 10 cm^2^, with the LD‐V1 positioned 600 mm from the x‐ray tube for mammography and 500 mm for the diagnostic x‐ray generator. To minimize scatter radiation, the non‐irradiated portions of the film were covered with 2‑mm‐thick lead shielding (Figure 2). Table 1 presents the exposure parameters. The effective energy data in Table 1 were obtained as described in Section B. One of the Methods. The mAs values were adjusted to achieve absorbed doses of approximately 1.0, 2.5, and 5.0 mGy. For mammography, a soft x‐ray chamber (type TN23344) was used, while for other x‐ray sources, a farmer chamber (type TN30013) was employed. The mAs values were initially estimated based on existing exposure data and prior dose measurements. These values were then fine‐tuned through preliminary measurements using ionization chambers to achieve absorbed doses of approximately 1.0, 2.5, and 5.0 mGy. The final mAs values were verified through repeated dose measurements to ensure accuracy. Additionally, the stability of the x‐ray output was confirmed with a coefficient of variation (CV) of less than 0.005 after 30 exposures, ensuring consistent performance.

**TABLE 1:** Exposure parameters by effective energies.

Effective energy [keV]	Tube voltage [kVp]	Tube current [mA]	Exposure time [s]	Additional filter	Absorbed dose [mGy]
18 (17.76)	28	25 [mAs]		–	1.02
		63 [mAs]		–	2.63
		125 [mAs]		–	5.27
30 (31.57)	65	100	0.071	–	1.04
		100	0.16	–	2.41
		200	0.16	–	4.94
50 (49.60)	120	50	0.08	4 mm Al	1.00
		100	0.1		2.60
		100	0.2		5.30
80 (81.31)	120	200	0.125	2 mm Cu	1.03
		200	0.32		2.68
		200	0.63		5.30

*Note*: The mammography system was set with irradiation conditions defined by mAs values.

#### 2.3.2 LD‐V1 scanning and analysis

The LD‐V1 was scanned using a flatbed scanner (EPSON DS‐G30000, Seiko Epson Co., Japan) in the RGB (48‐bit) mode at 100 dpi. To minimize image acquisition errors, particularly those related to the flatbed scanner light source, a sheet of regular white paper (Paper‐One Copier, APRIL Fine Paper, Indonesia) was attached to the back of the LD‐V1 for uniform pixel value. Additionally, to eliminate the non‐uniformity of the film layer, the LD‐V1 was scanned before and after exposure. To ensure consistent scanning conditions, the flatbed scanner was pre‐warmed before use to stabilize its light source. Specifically, the scanner was powered on at least 2‐h before use, followed by 10 preliminary scans to further stabilize the system. The tenth scan was used for analysis to minimize variations. The LD‐V1 films were scanned in a consistent orientation before and after irradiation to avoid alignment variations. Additionally, the scanning bed was cleaned before each scan, and the film was handled carefully using gloves to prevent artifacts caused by dust or scratches. To avoid pixel value changes due to buildup after LD‐V1 irradiation, the scan was fixed 24 h after exposure.

The image data of the LD‐V1 were divided into R, G, and B modes (16 bits each), and the R mode was used for high‐contrast pixel value analysis. The data were converted into grayscale and analyzed using ImageJ version 1.48v image analysis software (National Institute of Health, Maryland). To quantify radiation‐induced pixel value changes, the pixel values of the LD‐V1 before and after irradiation were subtracted in two dimensions across the entire scanned image. A circular region of interest (ROI) with a diameter of 30 pixels was then defined at the center of each irradiated region. Since the irradiated area was not visible in the pre‐irradiation image, the difference image (post minus pre) was used to identify the exposed field. The center of the square irradiation field was determined by drawing diagonals between its corners, and the ROI was centered at this point to ensure consistent positioning. Given the scanning resolution of 100 dpi (dots per inch), this corresponds to approximately 7.62 mm in physical size. This ROI size was chosen to ensure a representative measurement within the irradiated area while minimizing variations due to non‐uniformities at the film edges. The average NPV was calculated within this ROI using the following equation:

(1) $$\mathrm{NPV}=\frac{1}{N}\sum_{i=1}^{N}\left(P_{post,i}-P_{pre,i}\right)$$

where P*post*,i and P*pre*,i represent the red channel pixel values after and before irradiation, respectively, and N denotes the total number of pixels within the ROI. To quantify the uncertainty in the NPV measurements, the standard deviation (SD) of the NPV was calculated as follows:

(2) $$SD_{\mathrm{NPV}}=\sqrt{\frac{1}{N-1}\sum_{i=1}^{N}({\left(P_{post,i}-P_{pre,i)}-\mathrm{NPV}\right)}^{2}}$$

This equation represents the SD of the pixel value differences within the ROI, providing an estimate of measurement uncertainty.

The sensitivity of LD‐V1 was defined as the rate of change in NPV per unit dose, representing the film's response to the absorbed dose. Since the calibration curves were fitted to quadratic functions, the slope varies with dose. To quantify the sensitivity at different effective energies, the local slope of the NPV‐absorbed dose calibration curve was analyzed at three dose levels (10, 20, and 30 mGy). In this context, the local slope refers to the instantaneous rate of change of the calibration curve at a specific dose point. Since the curve is quadratic, this slope was obtained by calculating the first derivative of the fitted curve at each dose level. The relative sensitivity at each energy was calculated by normalizing the local slope to that at 30 keV using the following equation:

(3) $$\begin{array}{ccc} Relative\;Sensitivity & = & \left(\frac{Local\;Slope\;at\;energy\;E}{Local\;Slope\;at\;30\;keV}\right)\\ & & \times \;100\% \end{array}$$

This normalization allows for a comparative evaluation of the LD‐V1 response across different energy levels. The calculated relative sensitivity values, normalized to those at 30 keV, are presented in Table 2.

**TABLE 2:** Relative sensitivity (%) of LD‐V1 at different dose levels, normalized to 30 keV.

Absorbed dose [mGy]	18 keV	30 keV	50 keV	80 keV
10	52.8%	100%	92.6%	61.3%
20	56.8%	100%	97.2%	73.2%
30	63.8%	100%	105.0%	93.4%

The NPV‐absorbed dose calibration curves for each exposure parameter were then constructed using the NPV and absorbed dose values. These calibration curves were fitted to quadratic functions for each effective energy level. The slopes and coefficients of determination (*R* ^2^) were compared among the different effective energy levels to evaluate the response characteristics of the LD‐V1. Additionally, we assessed the relative response of the LD‐V1 at each effective energy level by calculating the relative NPV at each energy with respect to the NPV at the reference energy of 30 keV. This analysis allowed us to evaluate the energy dependence of the LD‐V1 across different diagnostic radiographic modalities. To quantitatively assess the energy dependence of the LD‐V1, the relative response was calculated by normalizing the NPV at each effective energy to the NPV at the reference energy of 30 keV, using the following equation:

(4) $$Relative\;Response=\frac{\mathrm{NPV}\;at\;energy\;E}{\mathrm{NPV}\;at\;30\;keV}$$

This normalization allows for a comparative evaluation of the LD‐V1 response across different energy levels. A relative response close to 1.0 indicates similar sensitivity to that at 30 keV, while deviations indicate variations in the film's energy dependence.

## 3 RESULTS

**FIGURE 3:**
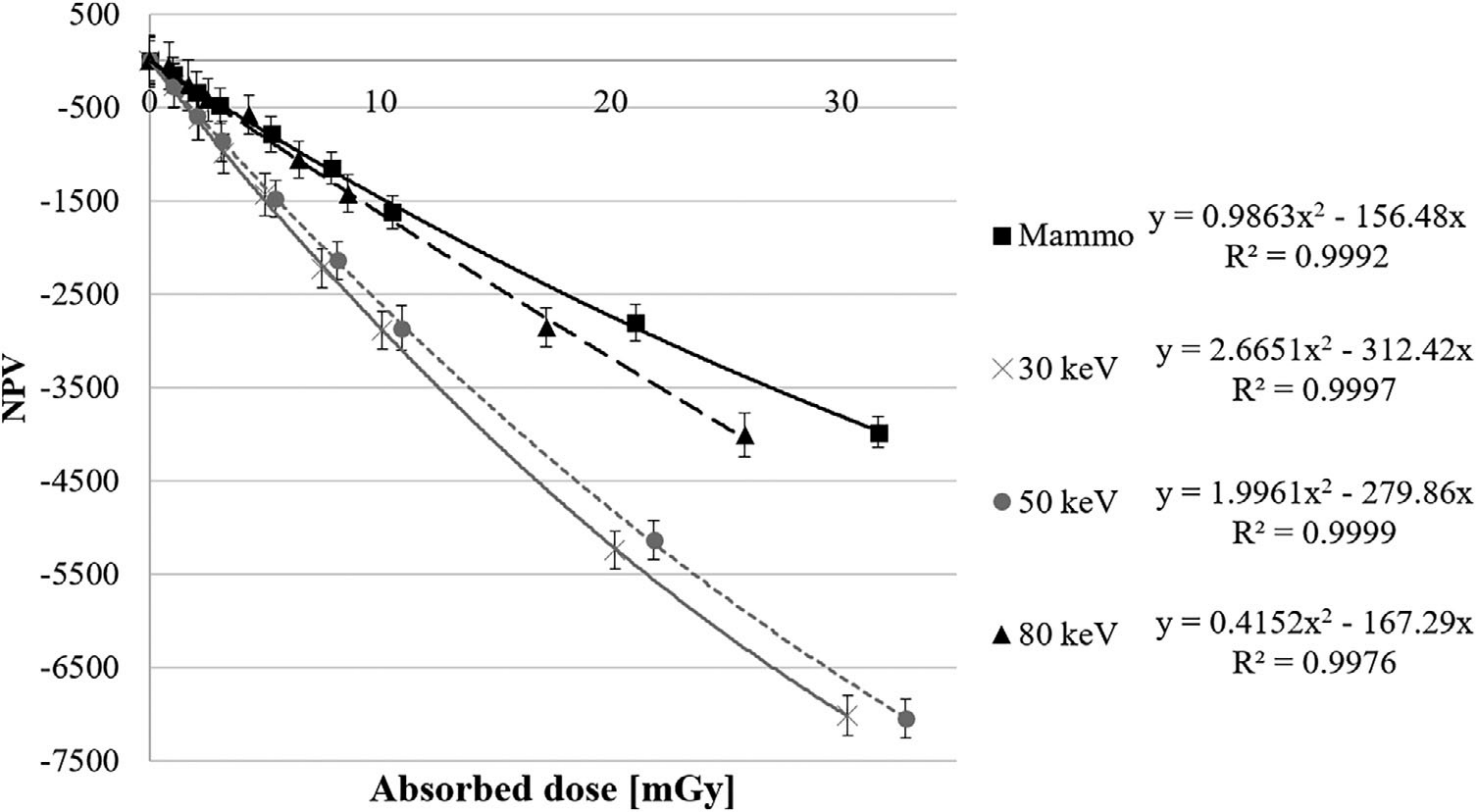
Calibration curve of NPV versus the absorbed dose for the LD‐V1. NPV, net pixel value; LD‐V1, GAFCHROMIC LD‐V1; *R* ^2^, coefficient of determination.

Figure 3 shows the NPV‐absorbed dose calibration curves for the LD‐V1 obtained at the four effective energies. The *R* ^2^ for the calibration curves at 18, 30, 50, and 80 keV were 0.9992, 0.9997, 0.9999, and 0.9976, respectively.

The local slopes at 30 and 50 keV were similar across all dose levels. The slopes at 18 and 80 keV were smaller than those at 30 and 50 keV. The quadratic fit was applied to all energy levels and represents the film response across the measured dose range.

**FIGURE 4:**
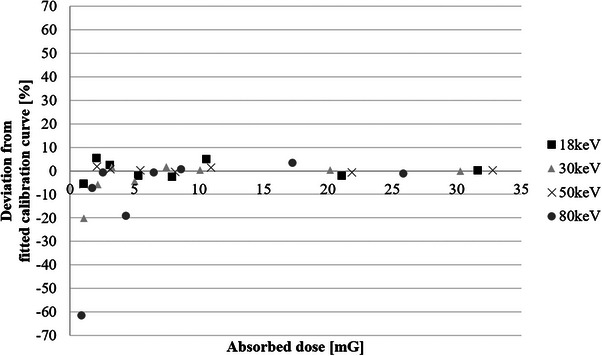
Deviation from the fitted calibration curves at different dose levels for each effective energy.

To assess the measurement accuracy at low to moderate doses, we analyzed the percentage deviation from the fitted calibration curves across the dose range of 1–30 mGy for each energy level (Figure 4). At 50 and 18 keV (mammography energy), the deviations were relatively small (−5.67% and −5.25%, respectively), suggesting that LD‐V1 provides reasonable accuracy at these dose levels. However, at 30 and 80 keV, the deviations were much larger (−20.03% and −61.27%, respectively), indicating a notable decrease in measurement precision at these energies. These results indicate that while LD‐V1 is sufficiently sensitive to detect doses as low as 1 mGy, careful consideration of measurement accuracy is required at this dose level. Generally, dose measurements with an error below 5% required doses of at least 3 mGy across all measurement conditions.

**FIGURE 5:**
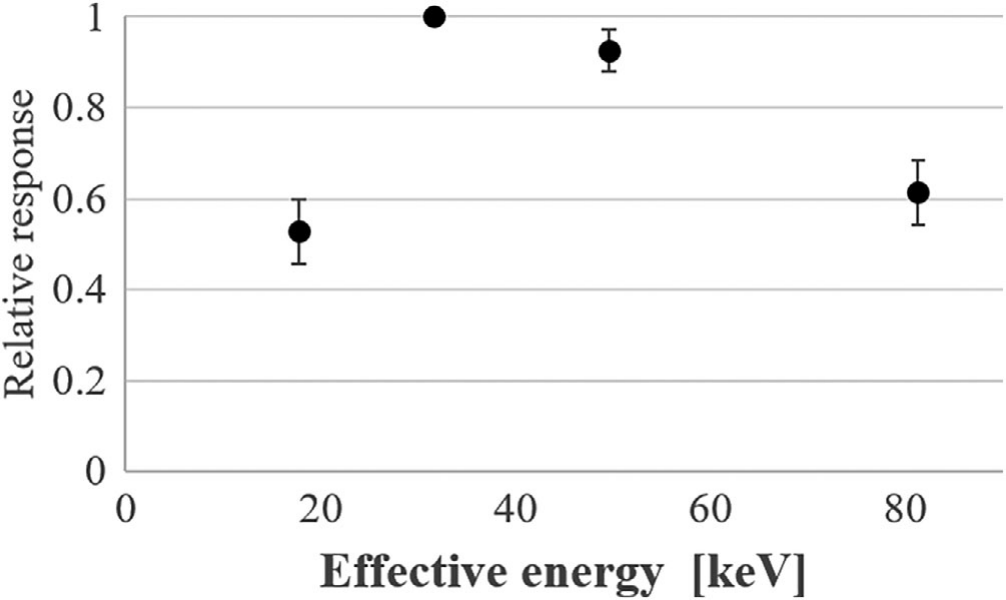
Relative response of the LD‐V1 for each effective energy when irradiated with 20 mGy based on 30 keV. LD‐V1, GAFCHROMIC LD‐V1.

Figure 5 shows the relative response of the LD‐V1 at each effective energy level, defined as the ratio of the NPV at a given energy to the NPV at 30 keV, after irradiation with 20 mGy. The calculated response values for mammography (18 keV), 50 keV, and 80 keV were 52.8%, 92.6%, and 61.4%, respectively, relative to the response at 30 keV (100%).

## 4 DISCUSSION

### 4.1 Energy dependence of the LD‐V1

Currently, LD‐V1 is the most sensitive radiochromic film available and would be of great benefit if it could measure low‐dose distributions as used in the diagnostic field. In this study, we investigated the response characteristics of the LD‐V1 at different effective energies used in diagnostic radiology. The manufacturer has set the recommended energy range to 40–160 kVp. Therefore, the response is likely low for energies lower than 40 kVp, including those used in mammography, due to the energy‐dependent interaction cross‐section of the LD‐V1's active layer. At lower photon energies, the probability of interaction through photoelectric absorption decreases, resulting in reduced sensitivity. This phenomenon has also been observed in other GAFCHROMIC films with similar compositions, such as the XR‐QA2, as noted in Reference 15. However, the LD‐V1 appears to have been optimized for a slightly different energy range, exhibiting even lower sensitivity in the mammography range compared to the XR‐QA2. We observed that the sensitivity at an effective energy of 18 keV (mammography) was approximately half that at 30 keV. Conversely, in the high‐energy region of 80 keV, assumed to be the maximum effective energy of CT systems, the response characteristic was approximately 60%, indicating significant energy dependence, as the response at 80 keV is approximately 60% of that at 30 keV. According to international dosimetry standards, the acceptable uncertainty for absorbed dose measurements in diagnostic radiology is typically within ± 10% (AAPM TG‐61, ICRU Report 87).^21^, ^22^ Given that the observed variation exceeds this threshold, this level of energy dependence could lead to substantial dose measurement errors if not properly accounted for. In CT, beam hardening from the bowtie filter causes variations in effective energy, potentially leading to dose underestimation. Based on our previous measurements^19^ the effective energy at the isocenter is approximately 50 keV, whereas, at a distance of 20 cm from the isocenter, it increases to 70 keV. This variation must be considered when calibrating LD‐V1 for CT dosimetry, as energy‐dependent sensitivity differences could affect dose measurements.

In this study, the bowtie filter was fixed to the CT system and could not be retracted during measurements. Consequently, the effective energy distribution varied depending on the position within the field of view, with lower energies concentrated near the isocenter and higher energies toward the periphery. This variation must be considered when calibrating LD‐V1 for CT dosimetry, as energy‐dependent sensitivity differences could affect dose measurements. Previous studies^16^, ^23^ have reported LD‐V1's nonlinear response at lower energies, consistent with our findings at 18 keV, and stable response between 30 and 50 keV. However, research on LD‐V1's energy dependence in the CT‐relevant range (50–80 keV) is lacking. Our study provides new insights by directly assessing its response in this range. LD‐V1's high sensitivity to low doses suggests potential applications in diagnostic dosimetry. In mammography, the mean glandular dose per exposure is typically in the range of 1–3 mGy, where precise dose assessment is crucial for balancing image quality and minimizing radiation exposure. In CT, low‐dose protocols are increasingly used to reduce patient radiation burden, with absorbed doses often falling below 10 mGy per scan. LD‐V1's ability to detect doses in this range highlights its potential as a dosimeter in these applications, provided appropriate calibration is performed.

The relative response of LD‐V1 exhibits a peak around 30 keV, followed by a decline at both lower and higher energies, which may be attributed to the influence of an absorption edge in the active layer. Although the exact composition of LD‐V1 remains undisclosed, its response characteristics suggest similarities with XR‐QA2, a diagnostic radiochromic film. However, the observed sensitivity of LD‐V1 is approximately half that of XR‐QA2, indicating potential differences in formulation. These findings underscore the necessity of dedicated calibration to account for energy‐dependent variations when utilizing LD‐V1 in diagnostic dosimetry.

### 4.2 Adaptability of the LD‐V1

The x‐rays used in mammography have a continuous energy spectrum; however, the application of a target filter generates x‐rays that approximate a line spectrum. Therefore, even if the LD‐V1 has an energy dependence in the low‐energy range below 20 keV, dose evaluation is possible by creating an NPV‐absorbed dose calibration curve tailored to the effective energy of the measured x‐rays. Additionally, our analysis of measurement accuracy suggests that dose measurements with an error below 5% generally require doses of at least 3 mGy across all energy levels. While LD‐V1 can measure doses as low as 1 mGy, its accuracy is highly energy‐dependent, with deviations as large as −20.03% at 30 keV and −61.27% at 80 keV. Therefore, when using LD‐V1 for dosimetry, it is necessary to irradiate doses of at least 3 mGy to ensure sufficient measurement accuracy. In addition to energy dependence, the reliability of dose measurements using LD‐V1 is influenced by several sources of uncertainty. The primary sources include dose measurement, scanning conditions, film uniformity, and x‐ray output stability. The uncertainty in dose measurement, which reflects the precision and calibration accuracy of the dosimeter, was estimated to be approximately ± 3%. Scanning‐related errors, due to slight variations in the light source and scanning position, also contributed an uncertainty of approximately ± 3%. Similarly, film uniformity introduced an uncertainty of ± 3%. The stability of the x‐ray output, confirmed through preliminary experiments, introduced a negligible uncertainty of ± 0.05%, with a CV of less than 0.005 after 30 exposures. When combined, these uncertainties resulted in an overall measurement uncertainty within ± 5%, ensuring the reliability of the calibration process and aligning with international standards such as AAPM TG‐61 and ICRU Report 87.

The sensitivity at 50 keV was comparable to that at 30 keV, with a relative difference of approximately 5–10% at 30 mGy, as shown in Figure 3. While this deviation exists, it falls within the general uncertainty range of ± 10% for dosimetric accuracy in diagnostic radiology (AAPM TG‐61, ICRU Report 87). Therefore, we consider this variation to be within an acceptable range, allowing for the use of a common calibration curve for dosimetry in general radiography and interventional radiology. However, for high‐precision applications, an independent calibration for 50 keV may be warranted.

In CT, the difference in effective energy between the center and the edge of the beam, ranging from 50 to 80 keV, is caused by beam hardening due to the bowtie filter. As most x‐ray irradiation is expected to be between 50 and 60 keV, the dose can be evaluated using the NPV‐absorbed dose calibration curve created at 50 keV. However, if the subject thickness is large and the field of view is sufficiently large to use the edge of the beam width, a high effective energy (70 keV or higher) may be used, which may cause a sensitivity reduction of 30%–40%.

## 5 CONCLUSIONS

This study demonstrated that LD‐V1 exhibits significant energy dependence, particularly at lower and higher photon energies. Using 30 keV (general radiography) as a reference, LD‐V1's relative sensitivity was approximately 50% at 18 keV (mammography) and 60% at 80 keV (the upper effective energy range for CT). These values were derived from the relative sensitivity equation presented in Section C.2., with detailed results provided in Table 2 and Figure 3. Given this energy dependence, careful calibration is necessary for diagnostic dosimetry applications. In mammography, the NPV‐absorbed dose calibration curve should be specifically adjusted to the effective x‐ray energy to ensure accurate dose assessments. In CT dosimetry, potential dose underestimation should be considered, particularly for larger patients exposed to effective energies exceeding 70 keV, where LD‐V1's sensitivity declines.

## AUTHOR CONTRIBUTIONS

*Concepts*: Gotanda Tatsuhiro. *Design*: Gotanda Tatsuhiro. *Definition of intellectual content*: Gotanda Tatsuhiro, Katsuda Toshizo. *Data acquisition*: Gotanda Tatsuhiro, Tomoyuki Hasuo, Shinnosuke Nishihara, Kohsei Matsuura. *Data analysis*: Gotanda Tatsuhiro, Tomoyuki Hasuo, Shinnosuke Nishihara, Kohsei Matsuura. *Experimental studies*: Gotanda Tatsuhiro, Tomoyuki Hasuo, Shinnosuke Nishihara, Kohsei Matsuura. *Literature search*: Gotanda Tatsuhiro, Tomoyuki Hasuo, Shinnosuke Nishihara, Kohsei Matsuura. *Manuscript preparation*: Gotanda Tatsuhiro. *Manuscript editing*: Gotanda Tatsuhiro. *Manuscript review*: Toshizo Katsuda, Yasuyuki Kawaji, Hidetoshi Yatake, Shinya Imai, Takuya Akagawa, Nobuyoshi Tanki.

## CONFLICT OF INTEREST STATEMENT

The authors declare no conflicts of interest.

## Data Availability

The data that support the findings of this study are available from the corresponding author upon reasonable request.
